# The Function of MicroRNAs in B-Cell Development, Lymphoma, and Their Potential in Clinical Practice

**DOI:** 10.3389/fimmu.2018.00936

**Published:** 2018-04-30

**Authors:** Bing Zheng, Zhijiang Xi, Rong Liu, Wei Yin, Zhiwei Sui, Boxu Ren, Heather Miller, Quan Gong, Chaohong Liu

**Affiliations:** ^1^Department of Immunology, School of Medicine, Yangtze University, Jingzhou, China; ^2^Clinical Molecular Immunology Center, School of Medicine, Yangtze University, Jingzhou, China; ^3^Wuhan Children’s Hospital, Tongji Medical College, Huazhong University of Science and Technology, Wuhan, China; ^4^Division of Medical and Biological Measurement, National Institute of Metrology, Beijing, China; ^5^Department of Intracellular Pathogens, National Institute of Allergy and Infectious Diseases, Bethesda, MD, United States; ^6^Department of Microbiology, School of Basic Medicine, Tongji Medical College, Huazhong University of Science and Technology, Wuhan, China

**Keywords:** B cell, microRNA, bone marrow, peripheral, germinal center, lymphoma, diffuse large B-cell lymphoma

## Abstract

B-cell formation, development, and differentiation are complex processes regulated by several mechanisms. Recently, there has been growing evidence indicating that microRNAs (miRNAs) are important for normal B-cell lineage development. miRNAs are small non-coding RNA molecules, about 20–22 nucleotide in length, that play an important role in regulating gene expression. They pair with specific messenger RNAs (mRNAs), resulting in mRNAs translational repression or degradation. Here, we review current research about the function of miRNAs in the aspects of B-cell physiology and pathology. We start by introducing the process of miRNA biogenesis. We will then focus on the role of miRNAs during B-cell lineage commitment and development in the bone marrow, followed by a discussion of miRNAs’ role in subsequent peripheral B-cell activation, proliferation, and final differentiation (including B-cell central tolerance and autoimmunity). We list and describe several examples to illustrate miRNAs’ role in the development of B-cell lymphoma, both as oncogenes and tumor suppressor genes. Finally, we delineate the potential value of miRNAs in diagnosing B-cell lymphoma, predicting clinical outcomes, and modulating the efficiency of anticancer treatments. Despite the vast amount of research conducted on miRNAs in recent years, it is still necessary to increase and further strengthen studies on miRNAs and their targets to promote a better understanding on B-cell development and as a result, construct more effective treatments against B-cell disease.

## Introduction

MicroRNAs (miRNAs) are small non-coding RNA molecules, generally 20–22 nucleotides long, that post-transcriptionally regulate gene expression in plants and metazoans (1, 2). miRNAs were first discovered in *Caenorhabditis elegans*. To date, about 2,500 human and 1,900 mouse miRNAs have been identified. Functional studies of miRNAs show that miRNAs regulate approximately 50% of all protein-coding genes. They are involved in most of the cellular physiological processes, including proliferation, development, and differentiation (3, 4). The changes of expression in miRNAs have been proven to be linked with many human pathologies.

Though miRNAs may be involved in several cellular physiological processes, do they regulate B-cell development? B-cell development includes several ordered steps, from the development of the B cell and its early stages in the bone marrow in the absence of an antigen to later stages that occur after interaction with the antigen in the periphery with the final result of antibody synthesis. Many investigators have studied the expression and function of miRNAs during B cell development, and evidence suggests that the answer to the question just presented is “yes.” Here, we provide a general overview of miRNAs’ roles during B-cell development in the bone marrow and in the periphery. In addition, we explore the function of miRNA expression in B-cell malignancies (Table 1).

**Table 1:** MicroRNAs’ (miRNAs’) role in B-cell development, central tolerance, autoimmunity, and lymphoma.

miRNA	Putative targets	Function	Reference
**Bone marrow B-cell development**			

miR-17-92 cluster	Bim	Essential for fetal and adult B-cell development; promote the survival of early B-cell progenitors (pro-B→pre-B)	(14, 15)
miR-34a	Foxp1	Inhibit B cell development at the pro-B to pre-B cell stage	(16)
miR-150	c-Myb, Foxp1	High levels in the lymph nodes, spleen, and thymus, premature expression block the transition from the pro-B to the pre-B stage	(17, 18)
miR-181	Unknown	Essential for B-cell lineage differentiation	(12)
miR-23a miRNA cluster	Trib3	Inhibit B-cell development	(20, 21)
miR-212/132	Sox4	Regulate the differentiation of prepro-B cells to pro-B cells and regulate B-cell survival	(22)

**Peripheral B-cell development**			

miR-146a	Numb	Regulate marginal zone B-cell differentiation	(30)
miR-155	Activation-induced cytidine deaminase	Regulate Ig isotype switching and somatic hypermutation	(31, 32)
	PU.1	Essential for germinal center response and high-affinity IgG1 production	(33, 34)
miR-125b	BLIMP-1 and IRF-4	Regulate B-cell differentiation in GC	(35)
miR-223	LMO2 and MYBL1	Regulate naïve to GC B cell and GC B cell to memory cell transition	(36)
miR-142	B cell-activating factor-R	Maintain B cell homeostasis; mice deficiency in miR-142 lead to increased size of splenic B cell compartment and hypoimmunoglobulinemia	(37)

**B-cell central tolerance and autoimmunity**			

miR-17-92 cluster	PTEN	Control B cell central tolerance at the immature B cell stage; Mice overexpressing the miR-17-92 cluster develop autoimmunity	(15, 44)
miR-148a	Gadd45a, Bcl2l11, and Pten	Regulate B cell central tolerance and autoimmunity, Increased miR-148a expression lead to lethal autoimmune disease in lupus mice	(45)
miR-210	Unknown	Mice deficient in miR-210 lead to production of autoantibodies; mice overexpressing miR-210 result in abnormalities in B cell subsets and function	(46)

**B-cell lymphoma**			

miR-17-92 cluster	Unknown	Accelerate tumor development in a mouse B-cell lymphoma model	(48, 49)
miR-155	TGFBR2, NIAM, histone deacetylase 4, SHIP1, and PIK3R1 (p85α)	Essential for cell proliferation, cell cycle, and apoptosis of diffuse large B-cell lymphoma (DLBCL) cell lines; acts as an oncogenic miR in B-cell lymphoproliferative disorders	(51–56)
miR-21	Unknown	Act as oncomiR to initiate tumor formation and maintenance	(61)
miR-217	Unknown	Promote the GC reaction; its overexpression promotes mature B cell lymphomagenesis	(62)
miR-181a	CARD11, NFKBIA, NFKB1, RELA/P65, and REL	Decrease cell proliferation and slower tumor growth rate by inhibiting NF-kB signaling activity	(63)
miR-34a	FoxP1, AXL	Inhibit the proliferation of various DLBCL cell lines; involve in the pathogenesis of DLBCL	(65, 66)
miR-146a	Early growth response-1 gene	Inhibit B-cell oncogenesis	(67, 68)
Cluster 15a/16-1 miRNAs	BCL2	Tumor suppressor in B-CLL and mantle cell lymphoma	(69, 70)
miR-28	MAD2L1, BAG1	Acts as a tumor suppressor in Burkitt lymphoma	(72)

## miRNA Biogenesis

MicroRNAs are processed from long hairpin-containing pri-miRNA transcripts. Pri-miRNAs are transcribed by RNA polymerase II and act as substrates for RNase III enzymes Drosha. In the nucleus, pri-miRNAs are cleaved by Drosha to generate pre-miRNAs with 70 nucleotides in length, and then pre-miRNAs are exported to the cytoplasm, in which, it is further processed into miRNA duplexes of ~22 bp long by Dicer. One strand of the duplex is the mature miRNA; this mature strand is usually incorporated into the RNA-induced silencing complex (miRISC). Argonaute (AGO) proteins, the main components of the miRISC, then directly bind with mature miRNA strands to cause subsequent gene silencing. GW182 proteins interact with the AGO protein to mediate this gene silencing. Finally, miRISC binds to its target messenger RNAs (mRNAs) and this result in their translation inhibition or degradation of the mRNA (5). A single miRNA can simultaneously mediate large numbers of different target genes. Likewise, a single mRNA can be regulated by multiple miRNAs (2, 4).

## B-Cell Development

B cells can be divided into B1 and B2 cell subclasses. B1 cells are components of the innate immunity and are characterized by a limited diversity of antigen receptors (6). B2 cells are conventional B cells and are involved in the adaptive humoral immune response. Going forward, B cells mentioned in this paper specifically refer to B2 cells. B-cell development initiates in the bone marrow with the asymmetric division of hematopoietic stem cells (HSCs). These HSCs then undergo successive differentiation to form common lymphoid progenitors that can further develop into either B cells or T cells. The majority of progenitors that remain in the bone marrow grow into B cells. Development of B cells continues in the bone marrow and peripheral lymph organs of an adult throughout his or her lifetime (7, 8).

Studies have shown that miRNAs play a crucial role in regulating the normal development of B cells. However, despite intensive study on miRNAs and B cells, the molecular pathways to regulate B-cell development is still not clear. Abnormal expression of miRNAs is considered to be closely associated with the pathogenic mechanism of B-cell malignant tumors, including mature and progenitor B-cell malignant tumors, such as non-Hodgkin lymphoma and B-lymphoblastic leukemia.

## miRNAs in Bone Marrow B-Cell Development

A defining feature in B-cell development is the rearrangement of genes in the immunoglobulin (Ig) loci, which results in the acquisition of a specific BCR on the B-cell surface (9). These rearrangements lead to the formation of immunoglobulin heavy (IgH) chains during the pro-B cell stage and immunoglobulin light (IgL) chains during the pre-B stage. The process of genetic random recombination of variable (V), joining (J), and diverse (D) gene segments, called V(D)J recombination, is mediated by RAG1 and 2 recombinases. At the end of this process, the immature B cell will acquire unique antigen specificity by expressing single IgH and IgL chains. Because the gene rearrangement is random, some pre-B cells will develop into immature autoreactive B cells. To remove or regulate autoreactive B cells, newly developed B cells will undergo the initial screening process through central tolerance mechanisms. If the B cell receptor can specifically recognize its self-antigen, it will undergo further V(D)J recombination at the IgL chain or the autoreactive B cell will die by apoptosis. Otherwise, non-autoreactive B cells will differentiate into mature B cells. Some B cells with self-reactivity can actually be further modulated through receptor editing in the periphery (10, 11).

To date, several miRNAs have been reported to act as antigen-independent modalities that are responsible for B cell development in the bone marrow. miR-181a was the first reported miRNA to affect B cell differentiation in the bone marrow (12). Researchers found that miR-181a was upregulated in the bone marrow, thymus, and spleen. Furthermore, overexpression of miR-181a by retroviral transduction in HSCs substantially increased B cell lineage in both tissue-culture differentiation assays and in mice (12).

The miR-17-92 cluster is composed of six single mature miRNAs (including miR-17, 18a, 19a, 20a, 19b-1, and 92a-1) and two paralogs: miR-106a~363 and miR-106b~25 (13). The miR-17-92 cluster is important for fetal and adult B-cell development. Ventura et al. used miR-17-92-deficient mice and found that B-cell development is inhibited at the pro-B to pre-B stage differentiation through modulating proapoptotic protein, Bim. Mice deficient in the cluster die shortly after birth from ventricular septal defects and lung hypoplasia (14). In another study, Lai et al. (15) were able to rescue the pro- to pre-B transition defect in miR-17-92 knockout cells using a lentiviral vector to express the miR-17 subfamily of the cluster. These studies show the miR-17 subfamily is important for early B-cell development (15), but discrepancy exists between these two reports (14, 15). The study performed by Lai et al. (15) found the expression of Bim in pro-B cell is only slightly increased in Mb1tKO mice (B cell-specific deletion of the miR-17-92 family miRNAs) compared with WT mice, so the finding excluded the role of Bim in mediating early B-cell development at the pro-B to pre-B transition, suggesting that some distinct molecular pathways and target genes should be involved in regulating miR-17-92 family miRNA control of B cell development. The pro-B to pre-B transition seems to be also mediated by another miRNA, miR-34a (16), transduction of the mouse bone marrow cell with a retroviral vector expressing both miR-34a and GFP resulted in reduced mature B cells. Further studies indicate that B-cell development is blocked at the pro-B to pre-B cell stage due to the involvement of miR-34a directly suppressing Foxp1, a B-cell oncogene (16).

miR-150 exhibits dynamic expression during B lymphopoiesis. Its expression is mainly in the spleen and lymph nodes; during B and T cell development, its levels greatly increase. Specifically, miR-150 expression increases strikingly at the immature B cell stage. When mouse bone marrow is transplanted with HSCs overexpressing miR-150 by a retroviral vector, the formation of mature B cells is extremely impaired. Additionally, the B-cell transition from the pro-B to the pre-B stage is blocked. Therefore, miR-150 might affect the pro-B and pre-B cell formation, a process regulated by targeting Myb and Foxp1, both of which are important for B-cell development (17, 18). miR-150 not only regulates the B2 cell development, but also affects the B1 cell formation in peripheral development and antibody response (17).

The miR-23a miRNA cluster includes three miRNAs: miR-23a, miR-24-2, and miR-27a. Its expression is regulated by the PU.1 transcription factor, which is indispensable for the immune cells’ differentiation from multipotential progenitors (19). Kurkewich et al. generated a miRNA-23a-,-24-2-, and -27a-deficient mouse model and observed that B lymphocytes in both the bone marrow and the spleen were significantly increased at the expense of myeloid cells (20). Increasing expression of miR-24-2 alone or the entire miR-23a miRNA cluster could simulate the function of PU.1 to facilitate hematopoietic stem cell differentiation into myeloid progenitors. This occurs at the expense of B cells both *in vivo* and *in vitro* (21). Therefore, miRNAs of the 23a cluster is also essential to regulate B cell lymphopoiesis.

The miR-212/132 cluster, identified in a recent study (22), has shown the ability to regulate B-cell development. In this research, B-cell development was inhibited when mice were transduced with a miR-132 overexpression vector. This inhibition occurred in the early B cell stage from prepro-B cell to pro-B cell. It was also found that the miR-212/132 cluster influences the survival of B cells. Another study proved that miR-132 regulates B-cell differentiation through inhibiting the transcription factor Sox4 (22).

The above data suggested that bone marrow B-cell development is a complex differentiation program and the process can be regulated by some miRNAs through targeting transcription factors, such as c-Myb, Foxp1, and Sox4 (16–18, 22). Different miRNAs showed positive or negative roles in regulating B-cell development, such that miR-34a, miR-150, miR-23a miRNA cluster and miR-212/132 inhibit early B-cell progenitor survival, whereas miR-181, miR-17-92 cluster promotes early B-cell differentiation from pro-B cells to pre-B cells. Undoubtedly, more miRNAs and their targets will be discovered to regulate the B-cell development in bone marrow, and miRNAs can mediate more complex gene expression.

## miRNAs in Peripheral B Cell Development

B-cell maturation occurs in the absence of antigen in the bone marrow and is then released into the periphery, where they re-circulate among the lymphoid organs, lymph, and blood. The B cells that have not been exposed to a specific antigen are called naïve B cells. Once naïve B cells are exposed to an antigen, some of the activated B cells (ABCs) directly differentiate into short-lived antibody-producing cells that mainly secrete IgM. The other B cells enter the follicle to establish a germinal center (GC) and eventually differentiate into high-affinity IgG-producing plasma cells and memory cells. The process of B-cell differentiation into plasma cells is regulated by activating the transcription factors Blimp1 an Xbp1 (23). GCs consist of three different regions that are termed dark zone, light zone, and mantle zone. The dark zone results from an intensive distribution of rapidly dividing B cells (centroblasts), whereas the light zone is made up of slower proliferating B cells (centrocytes) within the network of T follicular helper cells and follicular dendritic cells (DC). The non-ABCs are transferred to the border region of the follicle, forming the mantle zone. In the GC, B cells undergo Ig affinity maturation, where IgV genes are subjected to a series of somatic hypermutations, leading to differentiation into high-affinity antibody-producing plasma cells (24). Some autoreactive BCRs can be modified into non-autoimmune cells by a second V(D)J gene rearrangement. In addition, during the GC reaction, Ig genes undergo class switch recombination, and IgM constant regions are replaced by other Ig isotypes. This process results in generation of different effector functions of antibodies. Both somatic hypermutation and class switch recombination depend on the activity of activation-induced cytidine deaminase (AID) (25). Some centrocytes in the GC undergoing affinity maturation may eventually differentiate into long-lived memory B cells that can be reactivated when encountering the same antigen without the help of T helper (Th) cells (26, 27).

When the immature B cell arrives in the spleen, it develops into a marginal zone B cell (MZB) or follicular cell (FOB) (28). MZB cells are implicated in the early rapid response to infection by secreting IgM (29). To characterize miR-146a’s effect on B-cell maturation, King et al. used miR-146a-deficient mice to examine splenic B-cell subsets and found that MZB cells decreased in the spleen, while T1 and T2 transitional B cells increased. Therefore, miR-146a regulates MZB cell development by inhibiting Notch2 pathway through direct targeting of Numb in a T cell-independent (TI) manner (30).

miR-155 plays a key role in the mammalian immune system. It can negatively regulate AID (31, 32), whose function is mediating somatic hypermutation and Ig isotype switching. miR-155 is required for the B-cell response to T cell-dependent and TI antigens. It has been observed that the number of GC B cells is reduced in miR-155-deficient mice. Likewise, the number of GC B cells is increased with an enhanced antibody response in mice having an overexpression of miR-155 (33). B cells lacking miR-155 lead to a reduced GC response and failed secretion of class-switched, high-affinity IgG1 antibody (34).

To evaluate the function of miR-125b on B-cell differentiation, an LPS-treated B-cell line was transfected with a miR-125b mimic. B-cell differentiation into the plasma cell was inhibited as reflected by reduced IgM production. Further studies suggest that miR-125b regulates B-cell responses in the GC through targeting IRF-4 and BLIMP-1 transcription factors (35).

Zhang et al. performed B-cell miRNA profiling using multiplexed real-time PCR to observe the miRNA expression in the mature B-cell subsets. Researchers found that miR-223 expression is highly upregulated in both naïve and memory cells when compared with GC cells. By targeting transcription factors, LMO2 and MYBL1, miR-223 might regulate naïve to GC B-cell transition and GC B cell to memory cell transition (36).

In addition to the above miRNAs which regulate the B-cell development in peripheral, miR-142 has also been proven as a critical molecule to modulate the B-cell ontogenesis (37). In a miR-142 gene-deleted mouse model, the size of splenic B-cell compartment is enlarged, probably due to an immunoproliferative disorder that MZB cell population increased as well as B1 B cell decreased. Moreover, the function of B cells from miR-142 deficiency mice is impaired, manifested by hypoimmunoglobulinemia. And the B-cell homeostasis is controlled by miR-142 through targeting B cell-activating factor receptor (37).

In summary, several miRNAs have been implicated to affect the process of peripheral B-cell development and function, including MZB differentiation, GC response, Ig isotype switching, and somatic hypermutation. These processes are regulated by miRNAs through targeting different transcription factors that are important for antigen-dependent B-cell differentiation and function. Moreover, miRNAs expression profiling during B-cell activation would make us find some new molecules with critical role thus dissecting the profound function of miRNAs in peripheral B-cell development.

## miRNAs in B-Cell Tolerance and Autoimmunity

Immune tolerance is a state of unresponsiveness or low reaction of the immune system when encountering antigen. Immune tolerance ensures that the lymphocyte does not recognize its own tissues and only attacks “non-self” antigens. Defects in immune tolerance result in the development of autoimmunity and subsequently results in the attack of the body’s own tissues by autoreactive lymphocytes. Autoreactive B cells have been proposed as causative factors in several human autoimmune diseases. Examples include rheumatoid arthritis (RA) (38) and systemic lupus erythematosus (39, 40). In these two diseases, autoreactive B cells secrete proinflammatory cytokines and autoantibodies that damage the bodies own tissues (41). Although a number of in-depth studies have been conducted on the molecular mechanism of B-cell tolerance and autoimmunity, the role of miRNAs on B-cell tolerance is still unclear. Some studies have shown that miRNA expression in lymphocytes of patients with autoimmune diseases is generally different than those of healthy individuals (42, 43). The following miRNAs have shown the potential contribution to B-cell autoimmunity disease.

The miR-17-92 cluster was discovered as the first miRNA cluster to control B-cell tolerance. miR-19, one of the six mature miRNAs encoded by the miR-17-92 cluster, plays a critical role in regulating B-cell tolerance at the immature B cell. It inhibits the expression of PTEN, which functions as a negative mediator for the PI3K-Akt pathway (15). The role of miR-17-92 in mice was investigated by Xiao et al. Researchers found that transgenic mice with ectopic expression of miR-17-92 developed autoimmunity, producing higher serum autoantibody than control mice. These mice also died prematurely from the autoimmune disease at a higher rate than control mice (44).

Gonzalez-Martin et al. (45) performed a functional screen of individual miRNAs using IgMb-macroself mice transduced with a retroviral miRNA-expression library. They found miR-148a acts as a critical mediator to B-cell tolerance and autoimmunity. Three genes, Gadd45a, Bcl2l11, and PTEN, which are implicated in autoimmunity, were identified as miR-148a targets. Moreover, overexpression of miR-148a in lupus model mice can promote the development of a lethal autoimmune disease (45). Therefore, miR-148a may act as an important mediator for B-cell tolerance, as dysregulation of the miR-148a would lead to the development of autoimmune disease.

To investigate the role of miR-210 upon the B-cell activation and function, Mok et al. (46) generated miR-210 deficient and overexpressing mice and found miR-210, induced by Oct-2, act as a fine-tuning molecule to regulate the balance between pathogen elimination and autoimmunity. Mice deficient in miR-210 lead to the development of autoantibodies, whereas mice overexpressing miR-210 result in abnormalities in B-cell subsets and function, especially in decreased B2 cell population and impaired class-switched antibody production (46).

To our knowledge, except the above-mentioned miRNAs, very few studies have addressed the function of individual miRNAs for B-cell tolerance and autoimmunity. And these studies suggested that B-cell tolerance is possibly regulated by miRNA through mediating some molecules that are associated with autoimmunity, such as PTEN, Gadd45a, and Bcl2l11 (15, 44, 45). Although some reports have shown the miRNA expression profile in patients with autoimmune disease, the detailed role of miRNAs on B-cell tolerance is needed to be further explored.

## miRNAs in B-Cell Lymphomas

Abnormal expression of miRNA is common in some tumors, such as B cell neoplasms. B cell neoplasms develop from mature B cells and constitute the majority of lymphomas and leukemias. There is increasing evidence showing that miRNAs can function both as tumor suppressor genes and oncogenes (47). Many miRNAs are involved not only in the B cell development process, but also in B cell lymphoma development.

As discussed earlier, the miR-17-92 cluster is involved in bone marrow B-cell development and central tolerance. It also, however, shows oncogenic potential. Increased expressions of the miR-17-92 cluster cooperate with oncogene, c-myc, to promote tumor growth in a mouse model of B-cell lymphoma (48). Sandhu et al. generated miR-17-92 B cell-specific transgenic mice with an overexpression of miR-17-92. The penetrance of B-cell lymphoma in these mice was around 80% by age 12–18 months, suggesting that miR-17-92 is a strong cancer driver (49).

Though miR-155 has been demonstrated to function in both hematopoiesis and the immune response, it also acts as an oncogenic miRNA in many malignancies (50). Zhang et al. (51) used locked nucleic acid-modified anti-miR-155 to inhibit miR-155 expression in cultured BCWM1 and MEC1 cells *in vitro* and *in vivo* of a mouse xenograft model of Waldenstrom macroglobulinemia. Results showed an inhibition of cell survival and cell proliferation. Tumor growth in recipient mice also decreased, proving that miR-155 acts as an oncogenic miRNA in B-cell lymphoproliferative disorders (51). A recent study by Zhu et al. showed that expression levels of miR-155 were increased in lymphoma tumor tissues compared with adjacent normal tissues and those patients with lower miR-155 levels survived longer than those with higher levels (52). In addition, miR-155 has also been found to play an important role in cell proliferation, cell cycle, and apoptosis of DLBCL cell lines. It may regulate biological processes of DLBCL by targeting TGFBR2 (52). Some studies have also focused on the oncogenic role of miR-155 in the pathogenesis of B-cell lymphoma, including the targeting of NIAM, histone deacetylase 4 (HDAC4), SHIP1, and PIK3R1 (p85α) (53–56).

miR-21 functions as a proto-oncogene and contributes to cancerogenesis. miRNA profiling experiments indicate that its expression is upregulated in most tumor types analyzed so far, including human breast cancer, colon adenocarcinoma, and glioblastoma (57–60). Medina et al. have developed a mouse model using Cre and Tet-off technologies to overexpress miR-21. They found that ectopic expression of miR-21 can result in a pre-B lymphoma, which, therefore, suggests that miR-21 is an important oncogene (61).

A study presented by de Yebenes et al. (62) found that miR-217 acts as an oncogene in GC B cells. The mir-217 expression is upregulated in GC B cells and miR-217 can promote the GC reaction by stabilizing Bcl-6 expression, therefore, the production of class-switched antibodies and frequency of somatic hypermutation are enhanced. And miR-217 act as an oncomiR that its overexpression can promote the mature B cell lymphomagenesis (62).

In contrast to the oncogenic role of the mir-17-92 clusters, miR-155 miRNA, miR-21 miRNA, and miR-217 in B cell lymphomas, the following miRNAs that will be listed and discussed act as tumor suppressor genes. A study on miR-181a expression in GC B cell (GCB)- and ABC-like DLBCL cell lines and primary tumors showed lower miR-181a levels in the ABC-like subgroup compared with the GCB-like subgroup. This was found to be due to miR-181a directly regulating several components of the NF-kB signaling pathway and inhibiting NF-kB activity (63). When xenografts were performed, ABC-like DLBCL xenografts induced with miR-181a displayed a slower tumor growth rate and longer survival than GCB-like DLBCL xenografts. Overall, miR-181a negatively regulates oncogenic signaling which decreases cell proliferation and slows tumor growth rate (63).

miR-34a is a well-studied miRNA that acts as a tumor suppressor by linking with the p53 network in tumors (64). In an analysis of miRNA expression profiles from gastric MALT lymphoma and gastric DLBCL, 27 downregulated miRNAs were identified. Of the identified, myc-repressed miR-34a showed the strongest tumor suppressive activity in DLBCL cell lines. miR-34a’s tumor suppressive effects could be attributed to downregulate its target, FoxP1 (65). In another study, lymph node tissue was analyzed from 30 DLBCL patients and 30 reactive lymph node hyperplasia patients. miR-34a levels were significantly decreased in DLBCL lymph node tissue compared with control lymph tissue, demonstrating that miR-34a may be involved in the pathogenesis of DLBCL *via* its regulation on AXL (66).

miR-146a functions as a negative feedback regulator in the NF-κB pathway, where its deficiency in mice can result in the development of lymphoid and myeloid malignancies (67). Using the E(mu)-Myc transgenic mouse model, it was shown that miR-146a deficiency can synergize with c-Myc to form tumors and increase mortality in E(mu)-Myc mice. The early growth response-1 gene, which mediates hematopoietic differentiation, was identified to be regulated by miR-146a in B cells, suggesting that miR-146a also acts as a tumor suppressive gene to inhibit B-cell oncogenesis (68).

Cluster 15/16 miRNAs lie within chromosome 13q14 and are deleted or downregulated in B-cell chronic lymphocytic leukemias (CLLs). They also function as tumor suppressors through activating intrinsic apoptosis pathway by targeting BCL2 (69, 70). Expression of cluster 15/16 miRNAs is downregulated by c-Myc and histone deacetylase in mantle cell lymphoma cell lines. This is also observed in other Myc-expressing B-cell lymphomas (71).

Schneider et al. have investigated the role of miR-28 in B-cell lymphomagenesis (72). They compared the expression of miR-28 in normal and malignant GC B cells and found miR-28 expression is significantly decreased in Burkitt lymphoma (BL). Re-expression of miR-28 is able to reduce the proliferation and clonogenicity of BL cells; this re-expression also affects MYC-induced transformation in MCF10A cells through inhibition of MAD2L1 and BAG1. These results show that miR-28 is a tumor suppressor in BL and its downregulation leads to B-cell lymphomas (72).

Overall, previous studies demonstrated that the miRNAs in B-cell lymphomas can be divided into two groups: oncogenic miRNAs, such as mir-17-92 clusters, miR-155, miR-21, and miR-217 show the potential to initiate the tumor formation, maintenance and promote tumor development; whereas tumor suppressor miRNAs, such as miR-181a, miR-34a, miR-146a, Cluster 15a/16-1 miRNAs, and miR-28 exhibit the role to inhibit cell proliferation and tumor growth. In the future, it would be possible to control the expression of some key miRNAs and, therefore, develop some new therapeutic concept for the treatment of B-cell lymphomas.

## The Value of miRNAs in Diagnosis, Prognosis, and Therapeutic Potentials in B-Cell Lymphomas

MicroRNAs have been proven to mediate several cellular physiological activities and are closely associated with the formation, progression, and therapeutic response of several types of cancers, including B-cell lymphomas (73). An effective treatment currently used for DLBCL patients is rituximab, cyclophosphamide, hydroxyldaunorubicin (doxorubicin), oncovin (vincristine), and prednisolone regimen (R-CHOP).

Several miRNAs have been reported to affect the tumor sensitivity to specific drugs. For example, Marques et al. found that overexpression of miR-34a using a lentiviral vector can increase the sensitivity of two DLBCL cell lines to doxorubicin (74), and circulating miR-125b and miR-130a in DLBCL is associated with R-CHOP resistance (75).

MicroRNAs also have value as biomarkers for clinical diagnosis and prognosis in both CHOP- and R-CHOP-treated DLBCL cohorts (76, 77). Recently, Lawrie et al. examined circulating miRNAs in serum from both DLBCL and healthy controls, and found that the level of miR-21, miR-210, and miR-155 was higher in patients than controls. Therefore, these miRNAs may serve as diagnostic markers for DLBCL patients (78). Furthermore, another study investigated the early diagnostic value of circulating miRNAs in DLBCL. The authors measured levels of several miRNAs (including miR-15a, miR-16-1, miR-21, miR-29c, miR-34a, miR-155, and miR-223) in serum samples from DLBCL patients and healthy controls. In comparison with controls, analysis of these levels in DLBCL serum showed upregulation in miR-15a, miR-16-1, miR-21, miR-29c, and downregulation in miR-34a (79).

Researchers have also investigated the association of miRNA expression with clinicopathologic parameters and overall survival of patients. For instance, miR-21 is found to be overexpressed in peripheral blood mononuclear cells of B-cell non-Hodgkin’s lymphoma and serum of DLBCL patients. Higher expression of miR-21 is also positively associated with clinical stage and therapeutic outcomes, resulting in worse overall survival (80, 81). Conversely, another study evaluating the prognosis of miR-21 in Chinese DLBCL unexpectedly revealed that higher levels of miR-21 were consistent with better prognosis in DLBCL (82). Due to these differing results, the prognostic potential of miR-21 on DLBCL should be deeply explored in the future. Further study was performed to observe if miR-21 is able to regulate the cytotoxic effects of the CHOP chemotherapeutic regimen to the cell line CRL2631. Results showed that decreased miR-21 by antisense oligonucleotides can make DLBCL more sensitive to CHOP regimen (83). Similar results have shown that the miR-21 inhibitor can increase the sensitivity of DLBCL cells to doxorubicin (84). miR-21 can become a potential therapeutic target for B-cell lymphomas.

The value of miR-155 and miR-146a as potential prognostics in DLBCL patients has also been investigated (85). Zhong et al. analyzed the level of miR-155 and miR-146a in formalin-fixed tissue samples of DLBCL patients and found that their expression was higher in the DLBCL patients than in reactive hyperplasia lymphoid nodes. Further analysis showed that patients with lower expression of miR-155 and miR-146a exhibited longer progression-free survival in the *de novo* DLBCL. In addition, patients with high levels of miR-155 benefited more from rituximab therapy (85).

Another study performed by Mraz et al. (86) reveal miR-150 can act as the clinicobiological marker to reflect the outcome of CLL disease. It was found that miR-155 regulates two newly identified targets, Foxp1 and GRB2-associated bind protein 1, and both of which regulate the BCR signaling, therefore, affect CLL growth and survival. The CLL patients with higher levels of miR-150 in the blood showed longer treatment-free survival and overall survival (86). Interestingly, as mentioned before, miR-150 might affect the differentiation of pro-B cells to pre-B cells through regulating some potential targets, such as Myb and Foxp1 (18). However, Foxp1, as an essential regulator of early B-cell development (87), is not affected in miR-150 KO or overexpression of B cells; while Myb, an essential transcription factor, acts as the actual target of miR-150 in lymphocyte development (17). This is inconsistent with the findings of Mraz et al. (86) that Foxp1 expression from CLL cells is reduced in patients with relatively high-level compared with low-level expression of miR-150, whereas, Myb expression stay at the low level that is not proportional to miR-150. So it can be speculated that under different physiological and pathological conditions, the cell transcriptome would be different, thus, one single miR-150 can mediate multiple pathophysiological processes by targeting different molecules.

A similar study was performed by Wu et al. (88) to investigate the correlation of miR-146b-5p and the miR-320d expression on the prognosis of DLBCL patients. Results showed that their expression in lower levels was associated with poor clinical outcome, including reduced progression-free survival and overall survival. This is attributed to their inhibitory role in DLBCL cell proliferation (88).

Other studies focused on the relationship between expression of miRNAs and the prognosis of DLBCL patients. For example, low levels of miR129-5p in DLBCL patients have been associated with shorter overall survival (89), while high levels of miR-199a and miR-497 have been correlated with a better overall survival (90). Despite this, patients with high expression of miR-125b indicated poor prognosis along with shorter overall survival (75).

Overall, these studies demonstrated that miRNAs were increasingly recognized as a valuable tool for B cell lymphomas diagnosis, as prognostic markers to predict cancer clinical outcomes, and miRNAs even show the ability to modulate the efficiency in anticancer treatments. Thus miRNAs will show great potential in clinical practice.

## Concluding Remarks

**Figure 1:**
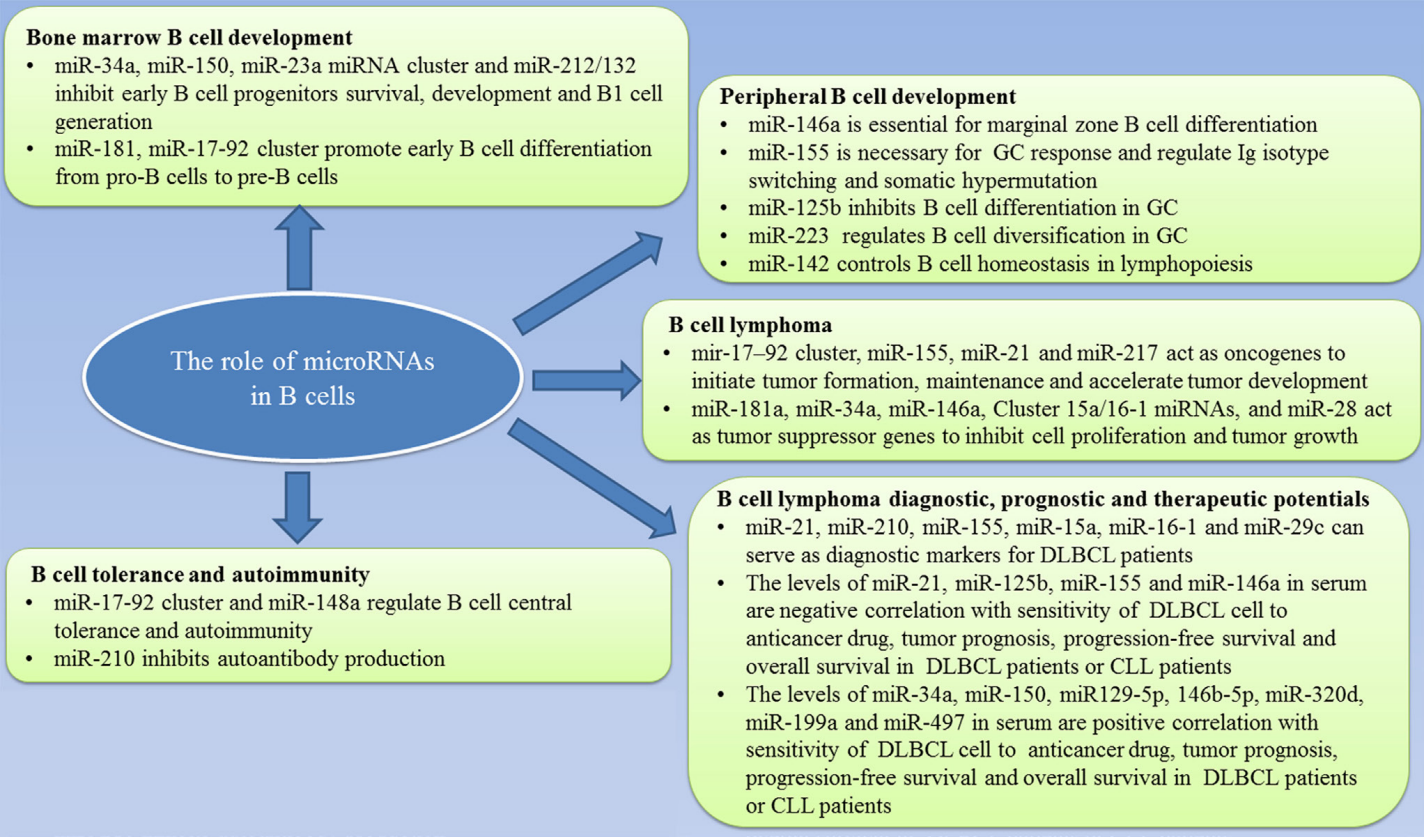
Summary of the role of microRNAs in B-cell development and lymphoma and the value in clinical practice.

MicroRNAs are a class of short, non-coding RNA molecules that negatively regulate gene expression by binding to their target mRNAs. The role of miRNAs in B cell physiology and pathology, and its potential in clinical practice are summarized in Figure 1. It is clear miRNAs also act as potent regulators of B cell development by regulating the gene expression of several transcription factors that are essential for B cell commitment, proliferation, and differentiation. B cell malignancies arise from the aberrant expression of proteins, processes of which have been shown to be regulated by miRNAs. However, the definite mechanism of how miRNAs affect the formation and biology of B-cell malignancies is not clear. To further clarify the function of miRNAs in B-cell malignancies, additional studies need to be conducted. Gain- and loss-of-function studies are ideal approaches that may be able to discover the information needed to jumpstart the use of miRNAs in clinical practice. In the future, more studies also need to focus on animal models for consistent and reliable results to enable us to better understand the pathophysiology of B cell malignancies.

In addition, various reports have suggested that miRNAs have the potential as non-invasive biomarkers for diagnosis, prognosis of B cell lymphomas, and even as targets in cancer treatment. Studies reviewed in this paper show that abnormal expression of miRNAs may help better assess patient responses to CHOP and R-CHOP chemotherapy regimens (and overall clinical outcome), which would supply us with better strategies to treat DLBCL. However, in order to further confirm the results and establish optimal cutoff values, it is essential to perform larger cohort studies. Furthermore, some miRNAs show the ability to regulate the sensitivity of DLBCL cells to R-CHOP. Therefore, combining the existing chemotherapeutic strategies with miRNAs might enhance the cytotoxicity of R-CHOP regimen, thus increasing the overall survival of DLBCL patients. So far, there are still few types of research on the detailed mechanisms of correlation in miRNA and R-CHOP effects. Further investigation is needed to clarify the roles of miRNAs in the modulation of R-CHOP treatment and investigate the prognostic potential of individual miRNAs in DLBCL patients. Finally, current reports are mainly conducted in DLBCL cells *in vitro*. More studies should focus on safety and efficiency of miRNA treatment in xenograft mouse models and in humans to ultimately make sure miRNAs are safe and useful for clinical practice.

## Author Contributions

CL organized the article. BZ wrote the draft. WY, ZS, BR, and HM revised the draft. ZX, RL drew the figure and table. QG edited the language, figure and table.

## Conflict of Interest Statement

The authors declare that the research was conducted in the absence of any commercial or financial relationships that could be construed as a potential conflict of interest.
